# Genomic Profiling and Mutation Analysis of *Mycobacterium bovis* BCG Strains Causing Clinical Disease

**DOI:** 10.3390/microorganisms13122853

**Published:** 2025-12-16

**Authors:** Benjamin Moswane, Olusesan Adeyemi Adelabu, Ute Monika Hallbauer, Morne Du Plessis, Jolly Musoke

**Affiliations:** 1 Department of Medical Microbiology, School of Pathology, Faculty of Health Sciences, University of the Free State, Bloemfontein 9300, South Africa; benjaminmoswane@gmail.com (B.M.); olusesanadelabu@gmail.com (O.A.A.); 2 Department of Paediatrics and Child Health, School of Clinical Medicine, Faculty of Health Sciences, University of the Free State, Bloemfontein 9300, South Africa; hallbute@ufs.ac.za; 3 Department of Genetics, Faculty of Natural and Agricultural Sciences, University of the Free State, Bloemfontein 9300, South Africa; duplessismg@ufs.ac.za; 4 National Health Laboratory Service, Department of Medical Microbiology, Universitas Academic Hospital, Bloemfontein 9300, South Africa

**Keywords:** tuberculosis, *M. bovis* BCG vaccine, whole-genome sequencing, BCG-associated disease, drug resistance

## Abstract

Tuberculosis remains one of the most prevalent infectious diseases, and the only currently available vaccine is the *Mycobacterium bovis* bacillus Calmette–Guèrin (BCG) vaccine. The uncontrolled passaging of the BCG strain led to genetically diverse BCG strains. Seven samples from clinical BCG-associated disease were obtained from the National Tuberculosis Reference Laboratory. Whole-genome sequencing and bioinformatics analysis were performed using tools such as fastqc, Trimmomatic, and CLC Genomics Workbench 24.0.3 to obtain consensus sequences and analyse deletions between *M. bovis* AF2122/97, BCG Danish, and clinical samples. Snippy was used to generate the phylogenomic tree, Prokka for annotation, and an in-house script to detect potential drug resistance. Four deletions were identified between *M. bovis* wildtype and *M. bovis* BCG. The phylogenomic tree showed that of the seven strains analysed, one was phylogenetically close to *M. tuberculosis* H37Rv, and another to the Danish BCG vaccine. Other samples were distantly related to each other and to reference strains. Two of the samples showed possible resistance to ethambutol. This would imply original misdiagnosis of the disease and subsequent ineffective treatment. This study emphasises the importance of genomic testing for accurate diagnosis of BCG disease and underscores the need for phylogenomic surveillance of *M. bovis* BCG strains circulating in South Africa.

## 1. Introduction

Tuberculosis remains one of the most prevalent infectious diseases, contributing significantly to global morbidity and mortality [1]. The only currently available vaccine against TB is the *Mycobacterium bovis* bacillus Calmette–Guèrin BCG vaccine [2]. The BCG vaccine is a live attenuated vaccine administered to children, particularly in countries with a high TB burden. The vaccine is generally safe. However, BCG-associated disease is a complication that can result from the administration of the vaccine [3]. BCG-associated disease can be localised (BCG-itis) or disseminated (BCG-osis).

The BCG vaccine was derived between 1908 and 1920 by attenuating the virulent wild-type *Mycobacterium bovis* strain on potato bile media [4]. The uncontrolled passaging after the distribution of the vaccine to different laboratories worldwide led to the development of genetically diverse BCG strains due to genetic drift [5]. This genetic variation affects both the effectiveness of BCG vaccination and the degree of severity of BCG disease [6].

Comparative genomic studies of the species in the *Mycobacterium tuberculosis* complex, such as *M. bovis* BCG and *M. bovis*, have shown regions of difference (RD), such as single-nucleotide polymorphisms (SNPs), tandem of duplications (DUs) and deletions [7,8,9,10,11]. The major attenuation of BCG in comparison to *Mycobacterium bovis* (*M. bovis*) is commonly linked to the deletion of the RD1 locus, which is absent in all BCG daughter strains and impacts the ESX-1 protein release pathway. The RD1 encodes for two secretory proteins, which are the culture filtrate protein (CFP-10) and the early secreted antigen target (ESAT-6), and they are important virulence factors. *M. tuberculosis* H37Rv requires RD1 to achieve complete virulence [12]. Deletion of RD1 in *M. tuberculosis* H37Rv has been demonstrated to result in strain attenuation [13,14]. Another study showed that strains that do not produce Phthiocerol dimycocerosate (PDIM) (i.e., BCG Moreau, BCG Glaxo and BCG Japan) are more attenuated [15].

However, BCG Pasteur supplementation with RD1 does not completely return virulence to wild-type levels [16]. In addition to RD1, BCG was discovered to have other genomic regions of difference (RD2 and RD3), and the specific locations and DNA sequences of each deletion were identified [5,17].

To the best of our knowledge, there has only been a single study reporting on the genomics of BCG in South Africa and there have been no published studies from any other region or country in Africa. Therefore, more studies must be conducted in this research niche [18]. This study aimed to determine genetic relatedness of BCG isolates and detect possible mutations using genomic profiling through whole-genome sequencing.

## 2. Materials and Methods

### 2.1. Ethical Considerations

Ethical approval was obtained from the Health Sciences Research Ethics Committee (UFS-HSD2023/1711/2805), Environmental & Biosafety Research Ethics Committee (UFS-ESD2023/0259) and the Free State Department of Health (FS_202309_012).

### 2.2. Study Design and Setting

This retrospective cross-sectional study involved retrieving BCG-identified isolates routinely submitted to the National TB Reference Laboratory (NTBRL) at the National Institute for Communicable Diseases (NICD). These isolates come from specimens submitted for investigation from clinical cases with BCG-associated disease. This study used comparative genomics to analyse the samples. The study was conducted at the Department of Medical Microbiology, Universitas Hospital, University of the Free State, Bloemfontein, Free State.

### 2.3. Sample Selection and Recovery

Seven stored BCG DNA samples isolated and identified between 2018–2023 using the standard operating procedure (SOP) that entailed the use of the Genotype Mycobacterium tuberculosis complex (MTBC) line probe kit were obtained from the National TB reference laboratory at the Centre for Tuberculosis, NICD. The seven BCG DNA samples were from BCG-associated cases from a setting where BCG Danish is routinely used for vaccination. Since the samples were obtained already in DNA form, no prior information about their processing was given. Due to the limited availability of samples, a convenience sampling approach was employed, using all seven available samples. The samples were transported for sequencing to the Agricultural Research Council (ARC) Biotechnology Platform according to the Dangerous Goods Regulation compliance guidelines, following regulations provided by the transportation of class 62 [19].

### 2.4. Whole-Genome Sequencing

Whole-genome sequencing was outsourced to the ARC and performed according to their SOP (as seen in Table S1), after which data analysis was on the raw sequences received. The process involved DNA fragmentation using a Covaris ultrasonicator (Covaris Inc., Woburn, MA, USA) with magnetic bead selection to obtain target fragment sizes, followed by end repair and A-tailing to prepare DNA ends. Adapter ligation was carried out with MGIEasy adapters, and the ligated DNA was cleaned using magnetic beads before PCR amplification. The amplified DNA underwent another cleanup step and was then denatured, circularised, and digested enzymatically to remove linear DNA. Following a final cleanup and quality assessment using Qubit assays, the prepared libraries were loaded onto MGI’s patterned flow cells and sequenced on the DNBSEQ platform (MGI Tech Co., Ltd., Shenzhen, China).

## 3. Data Analysis

The quality of the raw sequences was assessed using a software tool called fastqc (version 1.0) [20]. The sequences were then trimmed using trimmomatic (version 1.0) after running fastqc (version 1.0), and a command was used in which only the first 15 nucleotides were trimmed from the sequences (Headcrop: 15) [21]. Burrows–Wheeler Aligner (BWA) (version 1.0) was used for reference mapping, where the trimmed sequences were mapped individually against the reference genome (*Mycobacterium bovis*) [22]. SAMtools (version 2.0) was then used to filter mapped reads, keeping only those sequences that mapped to the reference, and all unmapped sequences were discarded. The mapped reads were sorted using SAMtools (version 2.0) to generate a sorted_reads binary alignment/map (BAM) file [23]. The BAM files of the seven samples (002, 007, 013, 015, 032, 034, 035) were then mapped against wildtype *M. bovis* AF2122/97 (GenBank accession: NC_002945.4) to generate consensus sequences using CLC Genomics Workbench (version 24.0.3) [24]. The consensus sequences were then extracted and exported back to the HPC cluster for further analysis. The obtained consensus sequences were then used for annotation in Prokka (version 1.0) [25].

The following steps were used to detect deletions between the *M. bovis* AF2122/97 (GenBank accession: NC_002945.4) and the BCG Danish strain used for vaccination in South Africa. Raw BCG Danish strain sequence was downloaded from the NCBI (National Centre for Biotechnology Information) with sequence read archive (SRA) number: SAMEA3257666 (https://www.ncbi.nlm.nih.gov/sra/, accessed on 15 February 2025). After downloading the sequence, fastqc (version 1.0) was performed on the sequence, and then trimmomatic (version 1.0) was used to trim the sequence. The first 12 nucleotides were trimmed (HEADCROP: 12), and then the trimmed output was used in CLC Genomics Workbench (version 24.0.3) for mapping to the reference, which was *M. bovis* AF2122/97 (GenBank accession: NC_002945.4). After mapping in CLC Genomics Workbench (version 24.0.3), the consensus sequence was then extracted as a fasta file and exported back to the HPC cluster to obtain the gaps in the consensus fasta file.

Additionally, the *M. bovis* AF2122/97 (Genbank accession: NC_002945.4) fasta file was used for annotation using Prokka (version 1.0) [25]. The deleted regions in the BCG Danish were located in the annotated general feature format (gff) output file. An in-house script was used to extract the region, start of feature, end of feature and the feature name.

A single nucleotide phylogenomic (SNPs) tree was constructed with a tool called snippy (version 1.1) and the consensus sequences of the seven samples were used together with BCG reference strains (BCG China, BCG Danish, BCG Frappier, BCG Glaxo, BCG Mexico, BCG Moreau Rio de Janeiro (RDJ), BCG Pasteur 1173P2, BCG Prague, BCG Russia, BCG Sweden, BCG Tokyo), *M. tuberculosis* H37Rv (GenBank accession: NC_000962.3). Two previously reported sequences in South Africa by [18], which are BCG_S48 (GenBank accession: CP033310) and BCG_S49 (GenBank accession: CP033311) and *M. bovis* AF2122/97 (GenBank accession: NC_002945.4) were used as the reference in snippy (version 1.1) [26]. A Python (version 3.13.0) script was used to loop over the samples to run them simultaneously. After running the samples, the snippy-core was used to generate a variant call file (VCF), and IQTREE (version 1.0) (https://github.com/Cibiv/IQ-TREE, accessed on 21 March 2025) was used to generate the phylogenomic tree, which was viewed using Figtree (version 1.4.4) (https://github.com/rambaut/figtree, accessed on 23 March 2025).

To identify potential antibiotic resistance-associated mutations, SNPs were extracted from Snippy (version 1.1) output files across the seven samples (002, 007, 013, 015, 032, 034, 035). These files were preprocessed using R version 4.3.2 [27]. The WHO mutation catalogue was also used to extract vital mutations that have been identified in the MTBC (https://www.who.int/publications/i/item/9789240028173, accessed on 29 March 2025). Data manipulation of the seven samples and the WHO catalogue was done using the tidyverse package (version 2.0.0), specifically readr for reading tab-delimited files and dplyr for data wrangling [28]. Gene names from the sample SNP data and the WHO mutation catalogue were standardised to uppercase. Amino acid positions were extracted from the AA_POS column in the SNP data files using regular expressions, with non-numeric or missing values filtered out. Similarly, entries from the WHO catalogue with defined amino acid mutation positions were retained and coerced into numeric format. A relational join was performed on gene names and amino acid positions to match variants present in the samples to those catalogued by the WHO as associated with drug resistance. This enabled the annotation of sample variants with relevant drug associations and WHO-assigned confidence gradings.

The raw sequences were submitted to the sequence read archives (SRA) on NCBI and the following bioproject number was assigned: PRJNA1332984. The samples were assigned the following accession numbers: SAMN51785509 (BCG Sample 002), SAMN51785510 (BCG Sample 007), SAMN51785511 (BCG Sample 013), SAMN51785512 (BCG Sample 015), SAMN51785513 (BCG Sample 032), SAMN51785514 (BCG Sample 034), and SAMN51785515 (BCG Sample 035).

## 4. Results

### 4.1. Annotation Summary of the Clinical BCG Isolates

The genome sizes of all seven clinical isolates were approximately 4.2 million base pairs (Mbp) (Table S2) The samples had the same number of tRNAs, rRNAs, tmRNAs, and repeat regions, except for one sample (Sample_035) which had one fewer tRNA. Furthermore, all seven samples (002, 007, 013, 015, 032, 034, 035) had coding sequences above 3900.

### 4.2. Deletions Between M. bovis AF2122/97, BCG Danish Vaccine Strain and the Clinical Isolates

Table 1 below depicts the presence or absence of deletions between wildtype *M. bovis* AF2122/97, BCG Danish vaccine strain and the clinical isolates. Three deletions in BCG Danish were absent in Sample 002 (regions 20, 29, and 47). Regions 15 and 20 were absent in Sample 034, and regions 20 and 47 were absent in Sample 035. Samples 007, 013, 015, and 032 all had all four deletions, although some regions were larger or smaller than the deletions located in the BCG Danish vaccine strain.

### 4.3. Annotated Genomic Features Present in M. bovis AF2122/97 but Absent in BCG Danish

Table 2 depicts the annotated genomic features within the genomic regions found in *M. bovis* AF2122/97 but deleted in BCG Danish. The first genomic region (1414059–1417217) had two genomic features: the ABC transporter ATP-binding protein and the serine/threonine-protein kinase. The second region (1769056–1778288) only contained hypothetical proteins, and the third region (2609507–2611482) also contained one feature: putative PPE family protein PPE40. The final region (4290995–4300496) contains features for the ESX-1 secretion system and also for the ESAT-6-like protein. These features are suggestive of RD1 as they are found in RD1.

### 4.4. Antibiotic Resistance-Associated Mutations

Two of the samples, as seen in Table 3 (002 and 015), were found to have mutations that potentially conferred resistance to ethambutol. Sample 002 had a missense mutation at nucleotide position 809 of the coding DNA (c.809) where a thymine (T) was replaced by a cytosine (C). Furthermore, the amino acid at position 270 (p.Ile270Thr) was changed from isoleucine (Ile) to threonine (Thr). Sample 015 had an adenine (A) nucleotide inserted between positions 1483 and 1484 of the coding DNA sequence (c.1483_1484insA), which resulted in a frameshift mutation. In addition, the amino acid sequence was altered after position 495, which was glycine (p.Gly495fs). The other five samples (007, 013, 032, 034, 035) did not have any mutations conferring drug resistance.

### 4.5. SNPs Phylogenomic Tree

**Figure 1:**
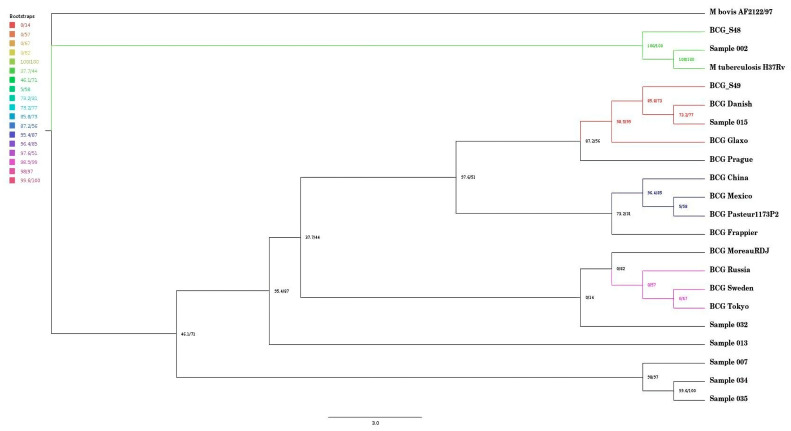
Single-nucleotide polymorphism phylogenomic tree of the seven samples and reference strains and previously sequenced isolates in SA.

Figure 1 presents a SNP-based phylogenomic tree of seven samples (002, 007, 013, 015, 032, 034, 035) alongside reference strains, including BCG China, BCG Mexico, BCG Pasteur 1173P2, BCG Frappier, BCG Danish, BCG Glaxo, BCG Prague, BCG Moreau RDJ, BCG Russia, BCG Sweden, BCG Tokyo, *M. bovis* AF2122/97, and previously sequenced BCG strains in SA (BCG_S48 and BCG_S49 and *M. tuberculosis* H37Rv. Four clades were identified, each indicated by a different colour. Sample 015 was closely related to BCG Danish and was on the same clade as one of the previously sequenced isolates in South Africa, which was BCG_S49. Samples 035 and 007 were distantly related to both the reference strains and the other samples. Notably, Sample 002 was closely related to *M. tuberculosis* H37Rv and clustered within the same clade as the other previously sequenced isolate, which is BCG_S48.

## 5. Discussion

This study aimed to detect genetic relatedness and possible mutations in seven clinical isolates (002, 007, 013, 015, 032, 034, and 035), identified as *M. bovis* BCG, using genomic profiling through whole-genome sequencing. Samples 007, 013, 015, 032, 034, and 035 exhibited identical genome sizes of 4,294,196 base pairs (bp), which was smaller than BCG genomes, such as BCG Danish 1331, which was shown to have a genome size of 4,411,814 bp by Borgers and colleagues [29]. The difference might be significant in the context of deletions as this might mean that our samples had more deletions than the BCG Danish strain used for vaccination in South Africa. The genome sizes of other members of the *Mycobacterium tuberculosis* complex (MTBC), such as *M. tuberculosis* H37Rv and *M*. *bovis,* are 4,411,529 bp and 4,345,492 bp, respectively [30,31]. However, Pan and colleagues reported similar genome sizes of approximately 4.2 Mbp for four BCG vaccine strains (BCG China, BCG Danish, BCG Russia, and BCG Tice) [32].

The isolates in this study had the same number of tRNA (52), tmRNA (1), rRNA (3) and repeat regions (2) except for isolate 035, which had one fewer tRNA (51). Modipane and colleagues also performed genome annotation of clinically isolated BCG isolates in South Africa and identified 45 tRNA genes, which is fewer than the 52 tRNA genes detected in our study. The fewer gene numbers in [18] was due to the use of draft genomes, hence the fewer tRNA genes as the genomes were incomplete. However, their study and this study reported an identical number of rRNA genes (n = 3) [18].

Sample 015 had identical deletions as BCG Danish, as seen in Table 1. The phylogenomic SNP tree (seen in Figure 1) also highlights the same observation, in that sample 015 was closely related to BCG Danish. This observation suggests that the patient had underlying conditions that increased susceptibility to the BCG vaccine complications itself rather than due to BCG vaccine strain mutations. The limitation of this study is that clinical information on the isolates was not available. However, a South African study has reported that most individuals who developed BCG-associated disease, such as BCG-itis had compromised immune systems [33]. A study from Iran also found that children who developed BCG disease often had an underlying condition, e.g., Mendelian susceptibility to mycobacterial disease (MSMD) [34]. Other conditions like severe combined immunodeficiency disease (SCID) and chronic granulomatous disease (CGD) are associated with susceptibility to BCG disease after vaccination [35,36,37]. Interestingly, Sample 015 was also on the same clade as BCG_S49, the previously sequenced isolate in SA, which also clustered closely with BCG reference strains like BCG Danish and BCG Glaxo [18].

The SNP phylogenomic tree showed that Sample 002 was closely related to *M. tuberculosis* H37Rv. The phylogenomic tree indicated that sample 002 was also on the same clade with a previously sequenced strain in South Africa (BCG_S48) [18]. The BCG_S48 in the study was found to be closely related to BCG Moscow and was on the same clade as *M. tuberculosis* EAI5/NITR2060 [18]. The result in our study indicated that this sample might have been misdiagnosed as *M. bovis* BCG instead of *M. tuberculosis*. Misdiagnosis can occur as a result of a flaw in the LPA assay used, or it could be because of the mutation occurring outside of the LPA’s target [38]. *Mycobacterium tuberculosis* H37Rv and *M. bovis* BCG belong to the MTBC, and differentiating them can be difficult [39]. This is because the mycobacteria belonging to the MTBC have a greater than 99% similarity due to the minimal variation in their core genes [40]. Routine diagnosis identifies MTBC. To distinguish MTBC specialised expensive tests such as polymerase chain reaction (PCR) and line probe assays are required [41]. Previous studies have reported the misdiagnosis of *M. tuberculosis* as BCG. A study by [42] initially indicated the misdiagnosis of tuberculosis as BCG; however, after DNA extraction and sequencing, the infant was confirmed to indeed have *M. tuberculosis*. Conversely, a study by Esteve-Sole and colleagues showed that it is possible to misdiagnose BCG as *M. tuberculosis* [43]. Another study indicated that the disease caused by BCG can clinically resemble active TB by causing systemic symptoms and involvement of organs such as the kidneys and lungs [44]. These study findings and previous reports highlight the importance of whole-genome sequencing in the accurate diagnosis of BCG-associated disease.

Sample 002 contained the region 4290995–4300496, which was found to have features that resemble RD1, which is essential for virulence [12]. However, BCG_S48, which was on the same clade as Sample 002 as can be seen in Figure 1, did not have RD1, which confirmed that it was *M. bovis* BCG [18]. In our study, Sample 002 contained the region with features that resemble RD1. Therefore, this might mean that our sample was not *M. bovis* BCG. The RD1 codes for the ESX-1 secretion system, which secretes two vital components, the ESAT-6 and the CFP-10 [45,46]. A previous study by Hsu and colleagues indicated that if the operons that code for the ESAT-6 and CFP-10 complex are disrupted, the cytolysis mechanism in macrophages and pneumocytes is disrupted and virulence is decreased [13].

Sample 015 had a frameshift mutation (seen in Table 3) caused by an insertion of an adenine between positions 1483 and 1484 of the coding DNA sequence, which resulted in the potential resistance to the drug ethambutol. A study involving BCG clinical isolates from patients in Argentina and Brazil found that one isolate was resistant to ethambutol based on culture-based drug susceptibility testing. However, no mutations associated with ethambutol resistance were identified in the genomic analysis [47]. Sample 002 was also found to contain a missense mutation (T → C), as can be seen in Table 3, which potentially could cause resistance to the drug ethambutol. This mutation might be a novel mutation as it has not been reported in the literature previously. Furthermore, since ethambutol is a first-line drug against TB disease and BCG-associated disease, this might have affected the treatment of the BCG disease case in our study. However, phenotypic testing is required to confirm the possible resistance observed. The other five samples did not contain any mutations conferring drug resistance.

The region from 4290995–4300496 was found to contain coding sequences that, upon annotation, revealed features typically associated with the RD1 region, such as ESX-1 secretion-associated proteins and ESAT-6-like proteins, as shown in Table 2. Sample 035 was found to have this region (4290995–4300496) as can be seen in Table 1. The presence of this region might be the reason why this isolate caused the disease. A previous study showed that RD1 is needed for full virulence of pathogenic *M. tuberculosis* [12]. Sample 034, on the other hand, had the first two regions according to Table 1 (1414059–1417217 and 1769056–1778288). The annotation of the first region (1414059–1417217), as shown in Table 2, revealed the presence of features such as the serine/threonine-protein kinase PknD and an ABC transporter ATP-binding/permease protein. The serine/threonine-protein kinase PknD was found previously to be vital to convey the virulence of pathogenic *M. tuberculosis*. In one study, the *M. tuberculosis* PknD was found to be necessary for tuberculosis disease that affects the central nervous system [48]. However, there is no evidence of the link between the pathogenicity of *M. bovis* and PknD. We observed putative deletions in several *PE/PPE* genes across the analysed samples. Due to the high GC content and repetitive nature of these regions, which can reduce coverage in short-read sequencing, these findings should be interpreted cautiously and would benefit from validation using long-read sequencing or targeted PCR.

The seven isolates obtained in this study were distantly related as seen in Figure 1. This was an interesting observation since the isolates were previously confirmed in the laboratory to be *M. bovis* BCG using PCR and line probe assay, which are standard tests used for diagnosis at the NHLS and NICD. The isolates were also all on different clades, and this shows that they might have developed different mutations over time and therefore different evolutionary lineages. Future investigations, such as transposon mutagenesis, are required to understand the functional implications of the observed mutations on bacterial adaptation and virulence. This could highlight the non-uniformity of *M. bovis* BCG genomes [49]. The findings could also mean that there are different *M. bovis* BCG strains circulating in South Africa. The implications can be critical, as that could mean that the population might have different levels of protection against TB, which might impact the protection against miliary TB or TB disease of the central nervous system among vaccinees. This is a concern, as the BCG vaccine currently in use in South Africa is the BCG Danish [50], and the expectation was for seven clinical isolates to cluster around the BCG Danish vaccine strain in the phylogenomic tree.

Due to the presence of both *Mycobacterium bovis* BCG and mixed sequences in the raw sequence data used in this study, reference-based mapping using BWA was performed instead of de novo assembly to ensure accurate alignment to the target genome. However, the reference mapping strategy can introduce reference bias since genetic variations between the sample and the reference genome might lead to misalignments, which could influence phylogenetic inferences and SNP variant calling [51]. Additionally, novel variations missing in the reference may be discarded [29].

## 6. Conclusions

High genetic diversity among the clinical *M. bovis* BCG isolates was observed in this study. Four clades were observed, one sample was closely related to the BCG Danish vaccine strain, one was potentially misdiagnosed as *M. tuberculosis* and the remaining was distantly related. These study findings suggest varied circulating *M. bovis* BCG strains in South Africa, which is of concern as the same vaccine, BCG Danish, is administered nationally. In addition, this study highlights the importance of using genomics for improved and accurate diagnosis of TB disease and especially BCG-associated disease. Furthermore, the findings of this study could be used for future surveillance and tracking the BCG disease and documenting the distribution of mycobacteria causing BCG-associated disease in South Africa. It is important to note that although WGS is a powerful tool, reproducible cultivation conditions are critical, and definitive demonstration of attenuation or virulence-conferring mutations requires genetic complementation studies.

## 7. Limitations of the Study

This study had limitations. Firstly, the clinical information of the patients from whom the samples were obtained was not available, therefore, the disease picture could not be linked to the genetic findings. Another limitation was the lack of information on the geographical origin of the samples and the age of the patients, which prevented us from studying or commenting on the demographic characteristics.

## Figures and Tables

**Table 1:** Presence or absence of deletions between *M. bovis* AF2122/97 and BCG Danish vaccine strain and the clinical isolates.

Region of Deletion in BCG Danish	*M. bovis* AF2122/97	BCG Danish	Sample 002	Sample 007	Sample 013	Sample 015	Sample 032	Sample 034	Sample 035
**15**	Absent	* Present (1414059–1417217)	Present (1413962 – 1417183) *	Present (1391328 – 1451201)	Present (1386360– 1462703)	Present (1413873–1417247)	Present (1120572– 1462703)	Absent	Present (1413633 – 1415032)
**20**	Absent	Present (1769056 –1778288)	Absent	Present (1767079– 1780031)	Present (1688405– 1781086)	Present (1768994–1778227)	Present (1623853– 1871385)	Absent	Absent
**29**	Absent	Present (2609507–2611482)	Absent	Present (2609162– 2611769)	Present (2586962– 2651528)	Present (2609378–2611393)	Present (2475295– 2680922)	Present (2609229– 2611787)	Present (2609780 – 2611408)
**47**	Absent	Present (4290995–4300496)	Absent	Present (4290674– 4293942)	Present (4282481– 4313921)	Present (4293850–4300393)	Present (4282482– 4313922)	Present (4298065– 4299603)	Absent

* Present (): The values in the parentheses indicate the locations of the deletions in the BCG Danish and the samples.

**Table 2:** Annotated genomic features within deleted genomic regions.

Region	Feature_Start	Feature_End	Feature_Name
1414059–1417217	1414258	1416654	ABC transporter ATP-binding/permease protein
1414059–1417217	1416665	1418395	Serine/threonine-protein kinase PknD
1769056–1778288	1769089	1769499	hypothetical protein
1769056–1778288	1769566	1770474	hypothetical protein
1769056–1778288	1770418	1771839	hypothetical protein
1769056–1778288	1771847	1772359	hypothetical protein
1769056–1778288	1772533	1773003	hypothetical protein
1769056–1778288	1773084	1773398	hypothetical protein
1769056–1778288	1773395	1773667	hypothetical protein
1769056–1778288	1773681	1774076	hypothetical protein
1769056–1778288	1774073	1774288	hypothetical protein
1769056–1778288	1774272	1775687	hypothetical protein
1769056–1778288	1775687	1776085	hypothetical protein
1769056–1778288	1776082	1776303	hypothetical protein
1769056–1778288	1776359	1776874	hypothetical protein
1769056–1778288	1776871	1778262	hypothetical protein
2609507–2611482	2610173	2612017	putative PPE family protein PPE40
4290995–4300496	4289559	4291334	ESX-1 secretion system protein EccCb1
4290995–4300496	4291477	4291773	PE family immunomodulator PE35
4290995–4300496	4291807	4292913	PPE family immunomodulator PPE68
4290995–4300496	4293006	4293308	ESAT-6-like protein EsxB
4290995–4300496	4293341	4293628	6 kDa early secretory antigenic target
4290995–4300496	4293753	4294154	hypothetical protein
4290995–4300496	4294816	4295742	ESX-1 secretion-associated protein EspI
4290995–4300496	4295811	4297274	ESX-1 secretion system protein EccD1
4290995–4300496	4297425	4298267	ESX-1 secretion-associated protein EspJ
4290995–4300496	4298325	4300559	ESX-1 secretion-associated protein EspK

**Table 3:** Mutations associated with antibiotic resistance in the samples.

Sample	Position	Type	Ref	Alt	Effect	Amino Acid Position Number	Drug	Final Confidence Grading
Sample 002	4176966	SNP	T	C	Missense variant c.809T > C p.Ile270Thr	270	EMB	Uncertain significance
Sample 015	4177640	InsA	G	GA	Frameshift variant c.1483_1484insA p.Gly495fs	495	EMB	Uncertain significance

EMB: Ethambutol; Ins: Insertion; SNP: single-nucleotide polymorphism. Ref: Reference; Alt: Alternate.

## Data Availability

The original raw sequences presented in the study were submitted to NCBI and are available with the following accession numbers: SAMN51785509 (BCG Sample 002), SAMN51785510 (BCG Sample 007), SAMN51785511 (BCG Sample 013), SAMN51785512 (BCG Sample 015), SAMN51785513 (BCG Sample 032), SAMN51785514 (BCG Sample 034), and SAMN51785515 (BCG Sample 035).

## Supplementary Materials

The following supporting information can be downloaded at: https://www.mdpi.com/article/10.3390/microorganisms13122853/s1, Table S1: Magnetic bead selection steps for a 100 μL sample to target specific fragment sizes; Table S2: Summary of the annotation details for the clinical isolates.
