# Supplementary material for: Genomic Profiling and Mutation Analysis of Mycobacterium bovis BCG Strains Causing Clinical Disease
Source: Microorganisms. 2025 Dec 16;13(12):2853. doi: 10.3390/microorganisms13122853 (PMC12735467; doi:10.3390/microorganisms13122853)
Supplement: Supplementary file 1 [file microorganisms-13-02853-s001.zip › microorganisms-3865326-supplementary.pdf]

Supplementary material

Table S1. Magnetic bead selection steps for a 100 µL sample to target specific fragment sizes.

**Target peak fragment size (bp)**	**180**	**230**	**280**	**335**	**420**	**550**
**1st bead selection (µL)**	**100**	**90**	**80**	**70**	**60**	**50**
**2nd bead selection (µL)**	**50**	**20**	**20**	**20**	**20**	**20**

Table S2. Summary of the annotation details for the clinical isolates.

**Clinical isolates**	**Genome size**	**Coding sequences (CDS)**	**tRNA**	**rRNA**	**tmRNA**	**Repeat regions**
Sample_002	4294208	3972	52	3	1	2
Sample_007	4294196	3976	52	3	1	2
Sample_013	4294196	3972	52	3	1	2
Sample_015	4294196	3971	52	3	1	2
Sample_032	4294196	3973	52	3	1	2
Sample_034	4294196	3974	52	3	1	2
Sample_035	4294196	3974	51	3	1	2
